# Efficacy of a high concentration of nano silver fluoride in preventing caries: A randomized controlled trial

**DOI:** 10.4317/jced.60776

**Published:** 2024-03-01

**Authors:** Juliane-Rolim de Lavôr, Rodolfo-Sinésio-Amador de Abreu, Samylla-Glória-de Araújo Costa, Débora-Heloísa-Silva de Brito, Thaysa-Gomes-Ferreira-Tenório Dos Santos, Nathalia-Kelly-Veloso-de Lima Almeida, Mabel-Cristina-Paiva-Machado da Silva, Arnaldo-de França-Caldas Júnior, Mônica-Vilela Heimer, André Galembeck, Aronita Rosenblatt

**Affiliations:** 1 DDS, MSc, PhD student. University of Pernambuco, School of Dentistry – Department of Pediatric Dentistry, Recife, Pernambuco, Brazil; 2 DDS, MSc. University of Pernambuco, School of Dentistry – Department of Pediatric Dentistry, Recife, Pernambuco, Brazil; 3 DDS, MSc student. University of Pernambuco, School of Dentistry – Department of Pediatric Dentistry, Recife, Pernambuco, Brazil; 4 DDS, MSc, PhD. University of Pernambuco, School of Dentistry – Department of Pediatric Dentistry, Recife, Pernambuco, Brazil; 5 DDS, PhD, Professor. University of Pernambuco, School of Dentistry – Department of Pediatric Dentistry, Recife, Pernambuco, Brazil; 6 DDS, PhD, Professor. Federal University of Pernambuco – Chemistry Department, Recife, Pernambuco, Brazil

## Abstract

**Background:**

To investigate the effectiveness of a novel agent containing Nano Silver Fluoride 1500 (NSF 1500) and chitosan to inactivate carious lesions in children.

**Material and Methods:**

The study included eighty children. While both groups had fluoride dentifrice applied to their teeth, only the experimental group received treatment with the NSF 1500-ppm solution. The first and sixth-month interval examinations were conducted by two calibrated dentists (k = 0.85).

**Results:**

The NSF 1500 group had 69.2% of their teeth with arrested decay, while the control group had 24.1%. The difference was statistically significant (*p* 0.001), with a preventive fraction of 59.4%. The number needed to treat (NNT) was approximately two. The NSF 1500 formulation was more effective than toothbrushing alone with fluoridated dentifrice in preventing dental caries.

**Conclusions:**

The effectiveness of NSF 1500 is determined by the size and depth of the dental cavity. Its ability to arrest caries lesions was comparable to previously tested products, NSF 400 and NSF 600.

** Key words:**Preventive dentistry, dental caries, nanoparticles.

## Introduction

The pandemic changed healthcare in many ways. Minimally Invasive Treatments have been the topic of many discussions, as they emerged as an alternative for removing tooth decay without using drills (1). Several products that prevent caries lesions progression and remineralize dental tissues have been proposed (1,2). These non-invasive interventions are usually easy to apply and low cost (3). According to the Global Burden of Disease (4) untreated dental caries is still one of the most common health conditions. If caries lesions are left untreated, they can lead to acute conditions, abscesses and sepsis, evolving into several systemic diseases (5). The most common barriers to children’s dental treatment are fear, financial constraints and a lack of access to health providers (6). When children suffer from acute caries and treatment is unavailable, it is necessary to employ low-cost caries control agents (7).

Products containing silver nanoparticles (AgNPs) are non-irritating and have excellent bactericidal properties against *Streptococcus mutans* biofilm (8,9). Because silver has a high chemical affinity for nitrogen, sulfur, and phosphorus compounds, its inhibitory power is derived from interactions with proteins with thiol groups and the phospholipid layer of the bacterial membrane (10). As a result, they can penetrate the bacterial cell wall, damage its membrane, prevent DNA replication/repair and inhibit the functions of its respiratory proteins (11). Nano Silver Fluoride (NSF) is a novel product previously studied by our research team that contains silver nanoparticles that safely stops the progression of carious lesions and have antimicrobial properties (12-14). This formulation differentiates because it includes: 1) fluoride, which reduces biofilm formation/adhesion and interferes with the demineralization/remineralization dynamics of dental enamel; and 2) chitosan, which chemically interacts with silver nanoparticles and increases its adhesion and stability by forming complex proteins and metal structures on dental hard tissues (15).

NSF treatment is simple, non-invasive and inexpensive. NSF, unlike Silver Diamine Fluoride (SDF), does not stain dental surfaces and doesn’t have a metallic taste (16). The efficacy of NSF in a randomized clinical trial (16) was comparable to SDF in a previous study conducted by the same authors under the same conditions (17). In both, after 12 months of a single application of each agent, the percentage of lesions treated that remained arrested was 66.9% for SDF (17) and 66.7% for NSF (16).

Therefore, this study aimed to assess the effectiveness of Nano Silver Fluoride 1500-ppm in preventing the progression of carious lesions. Also, to better understand the relationship between the lesions arrested, the number of affected tooth surfaces and the depth of the cavities.

## Material and Methods

This study was a double-blind randomized clinical trial (RCT) conducted in the city of Recife, Brazil, following the CONSORT (Consolidated Standards of Reporting Trials) guidelines. The research was carried out under the Helsinki Declaration and the Health National Council of Brazil (resolution 466/2012). Also, it was approved by the University of Pernambuco’s ethics committee (no. 08170412.0.0000.5207). Parents or legal guardians signed an informed consent to authorize their children’s participation in the study. The children included in the study signed informed consent as well. Both were written and explained in simple and appropriate language.

-Population and Sample Calculation

This trial was performed in Recife (Brazil), the largest city of the state of Pernambuco, with 3,995,949 inhabitants. The children enrolled in this RCT attended the University of Pernambuco Pediatric dentistry’s clinics. Initially, 319 children aged 5-7 years were examined. Of these, 80 children with at least one active dentin occlusal or proximal carious lesion in the primary molars (ICDAS 5), without signs or symptoms of pain or pulp infection, were included in the study. The teeth with both occlusal and proximal caries (occlusoproximal) were classified as proximal carious lesions; still, the same tooth with two or more separated cavities (occlusal and proximal), was also counted as proximal for data analysis at tooth and surface levels. A single trained investigator examined, selected, and treated the subjects. A different trained investigator worked on allocation concealment and randomization of the subjects.

The sample calculation was based on a previous pilot study with the solution tested in this RCT (NSF 1500 ppm) with a 6-month follow-up period. The estimated success rate for NSF 1500-ppm was 71.4%, while 26.2% for the control group. This calculation revealed that 44 teeth in each group were sufficient to generate statistically significant results, with type 1 errors ranging from 1 to 5% with a confidence interval of 95%. With an additional 20% of possible losses, the final sample calculation indicated 53 teeth per group. After baseline examination and recording of the decayed, missing, filled teeth (dmft) index, the children received computer generated random numbers inside opaque sealed envelopes that indicated the study group they were allocated to. Therefore, the randomization process was based on the subjects with an allocation ratio of 1:1.

-Preparation of Nano Silver Fluoride colloid

The nano silver fluoride colloid used in this study was patented under the number US20180280431 (https://patentscope.wipo.int/search) was synthesized as previously described.(18) An aqueous solution of AgNO3 (1.5 mL, 0.11 mol L−1) was added to a chitosan solution (30 mL, 0.01 g mL−1) previously dissolved in acetic acid (1% v/v) and filtered using a Millipore membrane (1.2 µm). The solution was transferred to an ice-cooled bath, and NaBH4 (1.5 mL, 0.66 mol L−1) was added dropwise under intense stirring (total Ag. = 5.54 x 10 3 mmol L−1).

Chitosan-stabilized AgNPs were then removed from the ice-cooled bath and stored in the dark. Aqueous solution of ascorbic acid (0.75 mL, 0.11 mol L−1) was added to chitosan solution in 1% acetic acid (30 mL, 0.01 g mL−1) under stirring. Different volumes of chitosan-stabilized AgNP seeds, recently prepared following the route described above, were then added. Finally, aqueous solution of AgNO3 (0.40 mL, 0.11 mol L−1) was added dropwise under vigorous stirring at room temperature. The resulting chitosan-stabilized AgNP colloids were stored in the dark at room temperature.(18) The silver nanoparticles had an average size of 3.2 ± 1.2 nm and a spherical shape. Fluoride (NaF) was added only at the end of the experiment, which improved the stability of the solution. The concentrations of each component, expressed in micrograms per milliliter, were as follows: Chitosan 28,585 μg/mL.; Ag+ 376.5 μg/mL., and NaF 5028.3 μg/mL..

-Clinical data collection 

Two calibrated dentists (interexaminer agreement k. = 0.85) examined and treated the patients, while two others were annotating data. The dentist who did not participate in the first exam performed follow-up inspections at six-month intervals. In either group, dentists did not remove unsupported enamel or dentin caries. On separate occasions, 10% of the sample was randomly selected to be rexamined for intra-examiner reproducibility. Intra-examiner reproducibility for caries diagnosis was calculated via Cohen’s kappa test, which indicated 0.90 for caries and 1 for arrested caries.

All participants received toothbrushes and toothpaste (1500 ppm of fluoride). Before clinical examinations and solution application, the children brushed their teeth to remove food debris. Then, they were subjected to a clinical inspection using LED lighting, dental mirrors, and dental excavators (WHO 1). Considering the caries treatment, no effort was made to remove the carious tissue or unsupported enamel. Cotton rolls were used to isolate the teeth from saliva. The NSF solution was in contact with the tooth surface for 2 minutes. Each tooth received two drops of 1500 ppm NSF with a microbrush, equivalent to a dose of 10 mg of the solution. The treatments were performed only once in 6 months. Nano-silver fluoride application protocol (14): 1) children brush their teeth to remove food debris; 2) children positioned seated on a regular chair; 3) isolate the decayed tooth with cotton rolls; 4) remove excess saliva with a gauze or cotton pellet, if needed, but no air drying; 5) apply the solution on the carious tooth surfaces with a microbrush and rub for 10 seconds; 6) wait 2 minutes to remove cotton rolls and conclude the application protocol; 7) inform the parent or tutor that the child should not eat, drink, or rinse mouth in the next 30 minutes.

The decayed, filled, and missing teeth (dmft) index is the most significant indicator used to measure oral health status (19). The dmft indexes and caries diagnostics were recorded based on the World Health Organization recommendations at 30, 90, and 180 days after NSF applications, with natural light and a ball-end probe. The exams were visual and tactile. If a wall or floor of the lesion was soft and easily penetrated by the probe using light force, then it was diagnosed as active. Arrested caries showed smooth, hard surfaces. Considering the child, decayed, missing, and filled tooth (dmft) index was chosen for recording dental caries experience. Considering the tooth, caries activity (arrested/active) and surfaces involved (occlusal and proximal) were the measurement.

-Statistical analysis

Data were analyzed using the software SPSS 20.0 for Windows (SPSS Inc., Chicago, USA). For the statistical analysis of the categorical data, descriptive statistics and Pearson’s Chi-square test were used. The continuous variables (dmft and age) were analyzed through means and standard deviation (SD). Dmft was also categorized (low and high) and analyzed through Pearson’s Chi-square test. The data normality assumption was checked through Kolmogorov– Smirnov test. The level of statistical significance was set at 0.05. Because the data was dichotomous (yes or no), the outcome took the rate of differences, the relative risk (RR), the relative risk reduction (RRR), the prevented fraction (PF), and the NNT into account.

## Results

**Figure 1:**
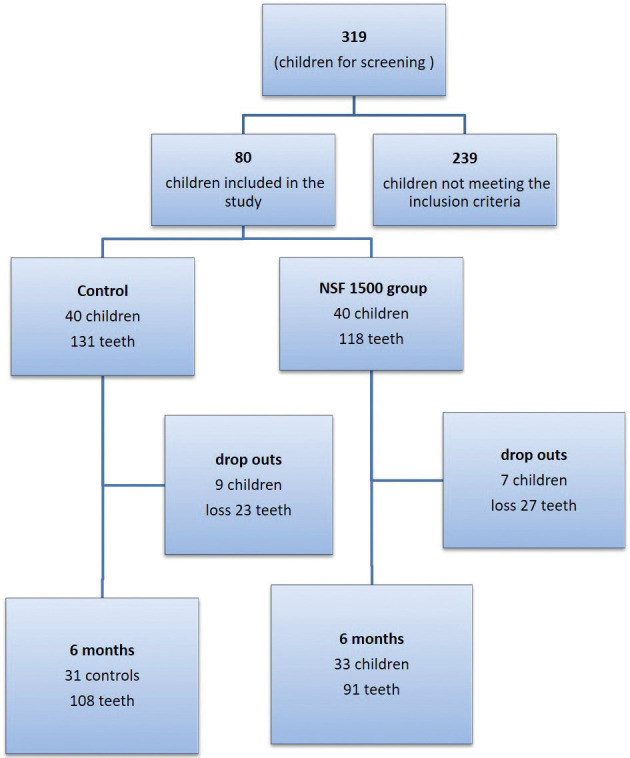
Sample inclusion, allocation and follow-up flowchart.

According to the flowchart (Fig. 1), 319 children had an oral inspection, and 80 were included in the trial. There were 16 dropouts at six months, a loss of 50 teeth. Table 1 describes the sample. Table 2 shows active caries and cavity types. The experimental product worked better in ICDAS 3 and 4 than in ICDAS 5 and 6. The results for caries arrest in the dental cavity were better in Class I than in Class II, which was statistically significant (Table 2).

**Table 1:**
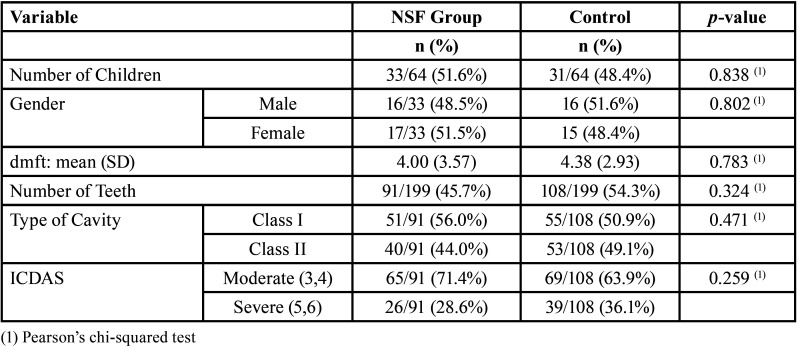
Sample characteristics of participants.

**Table 2:**
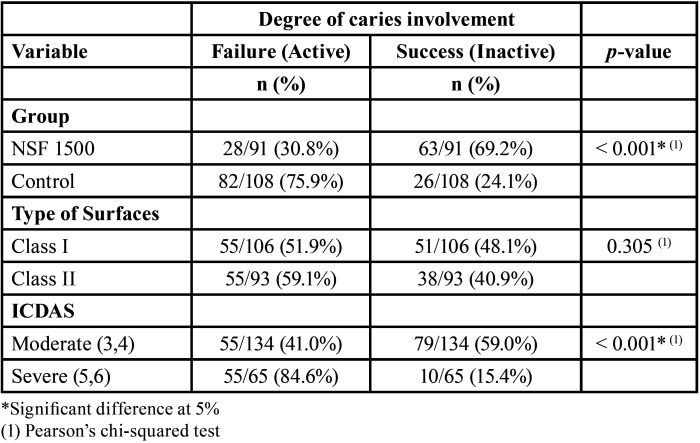
Assessment of caries at six months according to group, type of surfaces and ICDAS.

Caries had an RR of 2.47, nearly two and a half times higher than the NSF group. The PF was the RRR of the intervention’s risk reduction, which reduced the risk of tooth caries treated with NSF 1500 by 59.4%. The absolute risk reduction (ARR) represents the total reduction of NSF 1500-treated teeth compared to the control. The RRR, or PF, for the outcome of caries arrested, was 59.4%.

## Discussion

Non-invasive restorative techniques in deciduous teeth are becoming popular because they avoid drilling and filling of healthy dental tissues, reduce pain, and are well tolerated by pediatric patients (20). Composite resin restorative treatments require more precise techniques, take longer, and can be physiologically traumatic for children (21). Furthermore, due to the fragile dental structure of deciduous teeth, restoring complex cavities in primary teeth remains difficult for dental practitioners. Moreover, the composite resin has a high polymerization shrinkage, which can jeopardize marginal integrity (21), limiting the restoration’s longevity (20).

When deciding on dental treatment for children, the efficacy, effectiveness, costs, and the patient’s comfort and satisfaction with the treatment’s aesthetics should all be considered. While SDF is a non-invasive alternative treatment for carious teeth in children, it causes dental tissues to stain black, which may limit its global acceptance (22). Previous research on AgNPs found them effective agents for halting dental caries (23-25). The results of this study for the application of NSF 1500 were similar to those reported by Santos Jr. *et al*. (16), who evaluated NSF 400 in a similar trial design. This first clinical trial demonstrated that an AgNP-based formulation stopped caries in children, stopping 81% of dental decay after one week and 67% after 12 months.

For many years, the mechanisms underlying AgNP action were unknown. Recently, our team demonstrated, using scanning electron microscopy and focused ions, that AgNPs can penetrate dentin tubules and attach them to their walls. The slow release of Ag+ may explain the long-term carious stop effect of AgNPs observed in the current clinical trial and previous research (16,26). Andrade, Rosenblatt, and Galembeck (15) found that longer exposure times and smaller nanoparticle sizes increased the penetration depth of the AgNPs formulation used in this study. The total amount of silver found in intratubular dentin was estimated to be within the concentration range expected to inhibit bacterial growth while remaining low enough to avoid AgNP contact with the dental pulp. The AgNPs could inactivate the bacteria on the dentin surface by entering and lodging in its tubules. The constant source of ions (Ag+) prevents future bacterial invasion that could lead to caries development (22).

The presence of chitosan, which increases the viscosity of the solution and forms an AgNP colloid, is another advantage of the formulation containing AgNPs used in this study. Once SDF dissolves in water, the risk of a high amount of silver reaching the pulp expects to be much lower for AgNPs. The AgNP colloid containing chitosan can develop interactions with collagen fibers, as described by Rath *et al*. (27) and Hajji *et al*. (28). As a result, the nanoparticles can be trapped by the roughness of the tubule walls, preventing deeper penetration. Arnoud *et al*. (14) used a similar design to this trial to test NSF 400 and 600 and found a preventive fraction of 72%. These findings are consistent with laboratory research (29) on bacteria growth, which was directly affected by higher AgNP concentrations. However, in this trial, there were no significant differences between NSF 600 ppm and NSF 1500 ppm in preventing the progression of carious lesions.

AgNPs were measured from 8 to 10 nm in the current study, and AgNPs larger than 50 nm (massive) or charged (repelled by the electronegative character of the bacterial membrane) do not diffuse readily through the biofilm, according to Peulen and Wilkinson (30). After ten months of storage, the NSF 1500 ppm used remained sTable, and the product retained efficacy comparable to previous preparations. The small size of the nanoparticles (8-10 nm) and their spherical shape, which can penetrate the bacterial cell wall, causing direct and indirect lipid peroxidation, damaging the cell membrane, interrupting DNA replication, and inhibiting respiratory proteins, may explain NSF’s efficacy in caries arrest (23,24).

Hsiao *et al*. (31) found that glass-immobilized AgNPs outperformed silver-releasing substrates only in their ionic form for *E. coli* disinfection. However, the amount of silver in the solution was significantly higher than in suspensions or plates containing colloidal silver nanoparticles. As a result, AgNPs were more effective against *E. coli* than silver ions, implying that NSF is more effective than Silver Diamine Fluoride.

Targino *et al*. (23,24) compared the antimicrobial and cytotoxic activity of NSF, chlorhexidine, and SDF against *Streptococcus mutans*. NSF showed as a nontoxic bacteriostatic and bactericidal compound at any concentration for many erythrocytes types and was more biocompatible than SDF. NSF’s biocompatibility comes from the peptidoglycans, the main proteins involved with AgNP interactions. This structural feature of bacteria is absent in mammalian cells, meaning that AgNPs are toxic to bacteria but not humans (31). Thus, Xiu *et al*. (32) concluded that AgNPs do not exert significant direct toxicity to bacteria but may serve as mediators of Ag+ to bacteria up to the cytoplasm and membrane.

There is limited evidence that caries arrest treatment is effective in moderate caries lesions, such as those coded 3 or 4 in the International Caries Detection and Assessment System (ICDAS) (33). According to Duangthip *et al*. (34), the effects of topical fluoride on moderate caries lesions differed from those on cavitated dentine lesions. In this study, caries arrest rates in Class I cavities were higher than in Class II cavities, with the experimental product performing better in ICDAS 3 and 4 than in ICDAS 5 and 6, which was statistically significant.

It is critical to emphasize that the severity and extent of the caries lesion may exert an impact on the success of caries arrest treatment (34). Fung *et al*. (35) reported on four groups of children aged 3 to 4 years who received one or two applications per year, with varying time follow-ups, on the results of the most commonly used protocols for available caries stop treatments. The children in this study are six years old and, as a result, have more complex cavities than younger children because the first permanent molars have erupted and the dental arches have shortened, favoring the development of approximal caries. Nonetheless, the caries arrest rate at six months of NSF 1500 (69.2%) in the current study was higher than the results for SDF 12% (58.6%) and similar to SDF 38% (75.7%) reported by Fung *et al*. (35). NSF, like SDF, is simple to use, inexpensive, requires no invasive techniques, prevents cross-infection, and does not stain the tooth surface black.

## Conclusions

● The size and depth of the dental caries cavity are essential factors to the effectiveness of NSF 1500 ppm.

● Its ability to arrest caries was comparable to previously tested products, NSF 400 ppm and NSF 600 ppm, and did not stain the treated dental surfaces black.

● It may be an alternative of choice to increase access to dental care for uncooperative children and those with special needs.

● More research is needed to assess the lifetime storage of the highly concentrated colloid and develop new application protocols.
